# Supplementary material for: NLRP3 Inflammasome Activation in Hemodialysis and Hypertensive Patients with Intact Kidney Function
Source: Toxins (Basel). 2020 Oct 26;12(11):675. doi: 10.3390/toxins12110675 (PMC7693185; doi:10.3390/toxins12110675)
Supplement: Supplementary file 1 [file toxins-12-00675-s001.pdf]

Supplementary Materials: NLRP3 Inflammasome Activation in Hemodialysis and Hypertensive Patients with Intact Kidney Function

Christof Ulrich, Susann Wildgrube, Roman Fiedler, Eric Seibert, Leonie Kneser, Sylvia Fick, Christoph Schäfer, Silke Markau, Bogusz Trojanowicz and Matthias Girndt

Table S1. Medication of patients.

	HD	BP	*p* Value
ACE-I (%) (*n*)	5 (1)	15 (3)	0.298
ARB (%) (*n*)	70 (14)	60.0 (12)	0.507
Statin (%) (*n*)	30 (6)	30 (6)	1.000
ASS (%) (*n*)	30 (6)	25 (5)	0.702
Beta-Blocker (%) (*n*)	95 (19)	50 (10)	0.001
Diuretics (%) (*n*)	40 (8)	10 (2)	0.029
CC-Blocker (%) (*n*)	65 (13)	40 (8)	0.205
α_2-ARA (%) (*n*)	35 (7)	10 (2)	0.127

Abbreviations are: ACE-I, angiotensin converting enzyme-inhibitor; ARB, angiotensin receptor blocker; CC-Blocker, Ca channel-blocker; α2-ARA, α2-adreno receptor agonist. The differences in the two groups were analysed by paired Fisher's exact test.

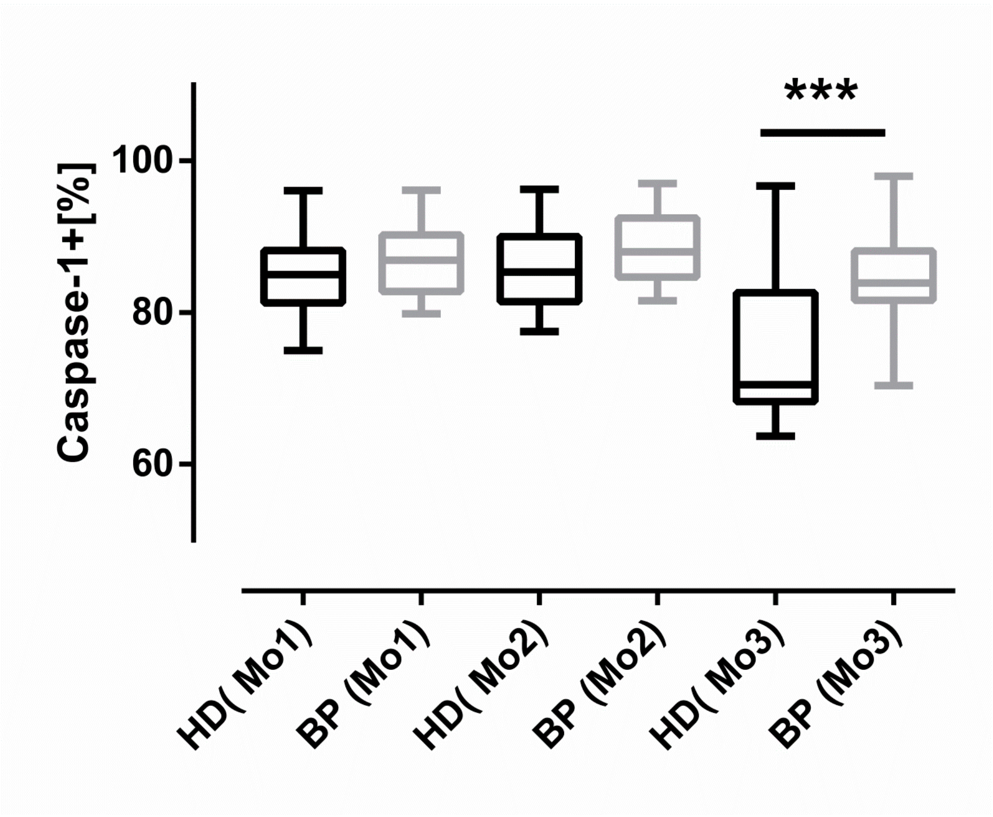

Figure S1. Frequency of monocyte subsets staining positive for caspase-1. Classical (Mo1), intermediate (Mo2) and non-classical monocytes (Mo3) staining positive for caspase-1 in haemodialysis (HD) and hypertensive patients with healthy kidney function (BP). The results are presented as box blots comprising median, 25th and 75th percentile. Statistical differences were analysed by One-way ANOVA, using Sidak's multiple comparisons test as post-test, *** $p < 0.001$.

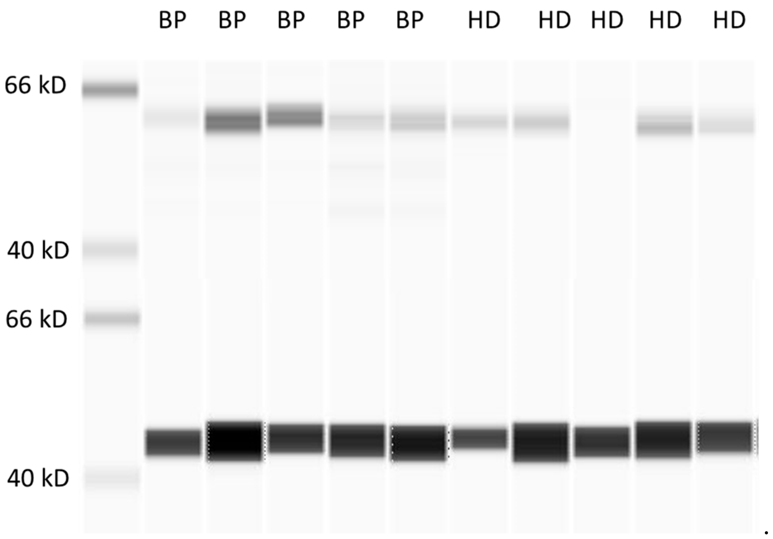
BP BP BP BP BP HD HD HD HD HD
66 kD
40 kD
66 kD
40 kD

(**a**)

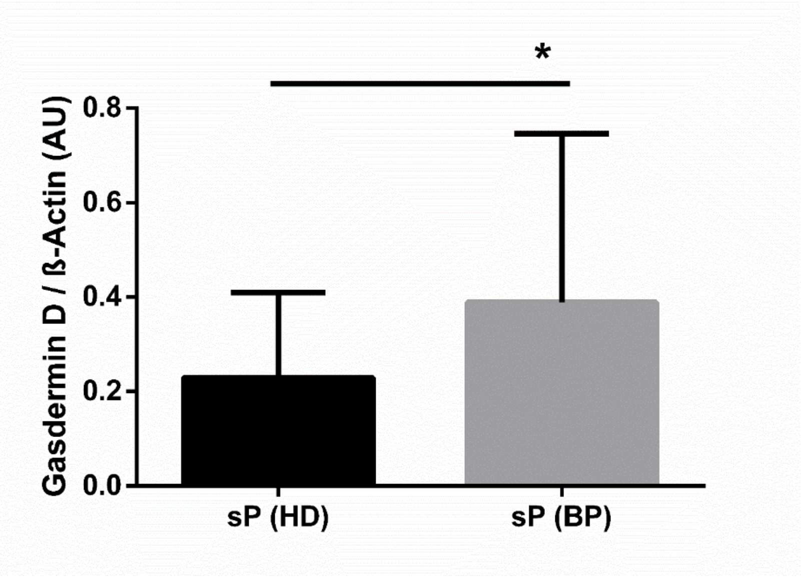
*
Gasdermin D / ß-Actin (AU)
0.8
0.6
0.4
0.2
0.0
sP (HD)
sP (BP)

(**b**)

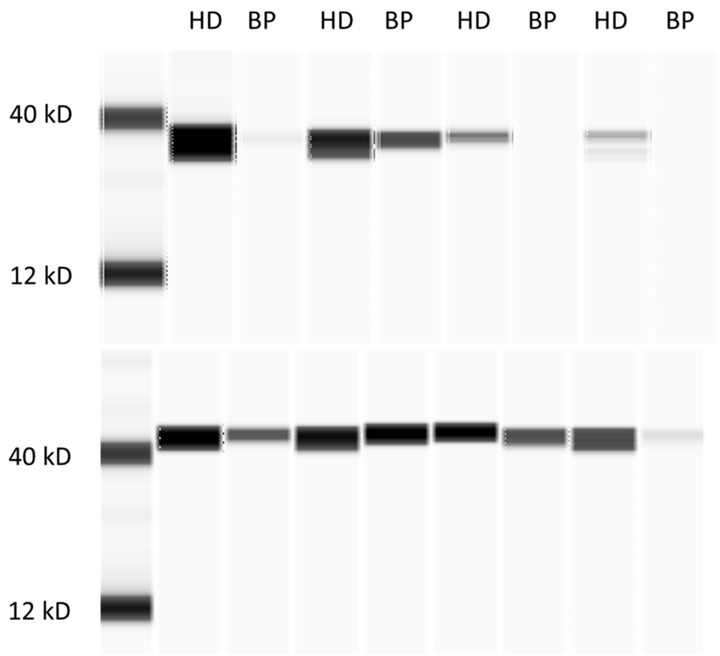

(**c**)

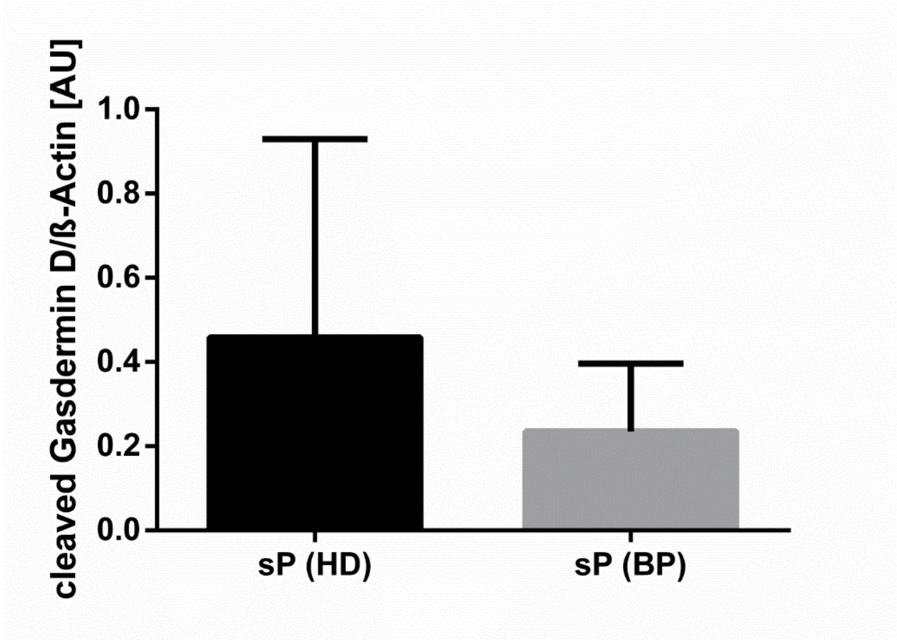

(**d**)

Figure S2. Analysis of gasdermin D (50 kD), cleaved-gasdermin D (30 kD) and β-actin (43 kD) in PBMC (P) of hemodialysis (HD) and overweight hypertensive patients (BP) under stimulatory conditions (s); (**a**) exemplary capillary electrophoresis blot of cell lysates analysing gasdermin D of BP and HD patients; (**b**) gasdermin/β-actin ratio in HD and BP patients; * $p = 0.05$; (**c**) exemplary capillary electrophoresis blot of cell lysates analysing cleaved- gasdermin D of BP and HD patients; (**d**) cleaved-gasdermin/β-actin ratio in HD and BP patients.
